# Single and combined exposure to ‘bee safe’ pesticides alter behaviour and offspring production in a ground-nesting solitary bee (*Xenoglossa pruinosa*)

**DOI:** 10.1098/rspb.2023.2939

**Published:** 2024-03-20

**Authors:** Sabrina Rondeau, Nigel E. Raine

**Affiliations:** ^1^ School of Environmental Sciences, University of Guelph, Guelph, Ontario, Canada N1G 2W1; ^2^ Department of Biology, University of Ottawa, Ottawa, Ontario, Canada

**Keywords:** flupyradifurone, field-realistic exposure, ground-nesting bees, SBI fungicide, squash bee, synergy

## Abstract

Mounting evidence supporting the negative impacts of exposure to neonicotinoids on bees has prompted the registration of novel ‘bee-friendly’ insecticides for agricultural use. Flupyradifurone (FPF) is a butenolide insecticide that shares the same mode of action as neonicotinoids and has been assessed to be ‘practically non-toxic to adult honeybees' using current risk assessment procedures. However, these assessments overlook some routes of exposure specific to wild bees, such as contact with residues in soil for ground-nesters. Co-exposure with other pesticides may also lead to detrimental synergistic effects. In a fully crossed experiment, we assessed the possible lethal and sublethal effects of chronic exposure to two pesticides used on *Cucurbita* crops, the insecticide Sivanto Prime (FPF) and the fungicide Quadris Top (azoxystrobin and difenoconazole), alone or combined, on solitary ground-nesting squash bees (*Xenoglossa pruinosa*). Squash bees exposed to Quadris Top collected less pollen per flower visit, while Sivanto-exposed bees produced larger offspring. Pesticide co-exposure induced hyperactivity in female squash bees relative to both the control and single pesticide exposure, and reduced the number of emerging offspring per nest compared to individual pesticide treatments. This study demonstrates that ‘low-toxicity’ pesticides can adversely affect squash bees under field-realistic exposure, alone or in combination.

## 1. Introduction

The pressing need to investigate the potential for combined effects from exposure to co-occurring pesticides in aquatic and terrestrial ecosystems has driven extensive research into pesticide mixtures, with global assessments across watercourses, soil, and plant and animal tissues consistently revealing widespread pesticide detections, predominantly in mixtures [1–3]. Numerous experiments have explored pesticide mixture effects on a range of animal taxa [4,5], often using overly simplified conditions that diverge significantly from real-world environments. Typically conducted in laboratories, these experiments primarily focus on acute toxic exposure while neglecting comprehensive assessments of chronic exposure and possible sublethal effects [6]. Despite the evident advantages that field and semi-field research can offer to the field of ecotoxicology, such studies remain scarce.

Pesticide use is an important driver of documented insect and bee declines [7,8], threatening biodiversity, agricultural production and food security. Current knowledge about the effects of pesticides on bees comes from research focused on the consumption of contaminated pollen and nectar by a limited number of eusocial species, namely honeybees (*Apis mellifera*) and bumblebees (*Bombus* spp.) [9–11]. However, variations in pesticide exposure and sensitivity across bee species, attributed to differences in physiological and life-history traits [12], underscore the necessity of exploring diverse exposure routes to gain a better understanding of the full impacts of pesticides on wild bee communities. Over 80% of bee species in North America [13], including 65% of those associated with key pollinator-dependent crops like apple and blueberry [14], nest underground and may be at risk of chronic contact exposure with pesticide residues in soil during nest construction and immature development [15,16]. Many of these ground-nesting bee species are solitary [14], heightening their vulnerability to pesticide exposure due to variation in life-history traits such as foraging specialization, foraging season duration, body size, food provisioning behaviour and the absence of colony-level buffering effect [9,12].

Over the past two decades, a class of widely used neurotoxic insecticides, the neonicotinoids, has increasingly been linked to negative effects on bees [10,17]. Neonicotinoids act upon the central nervous system of insects as agonists of the nicotinic acetylcholine receptors, causing neuronal hyperexcitation and death [18]. Being highly persistent and systemic insecticides, neonicotinoids commonly contaminate the flower, soil and water resources that bees rely upon [15,19–21]. Sublethal effects of neonicotinoids on bees often manifest in impairment of learning and memory functions, foraging behaviours, motor activity, and orientation and navigation (reviewed in [10,22]). In ground-nesting bees and other wild bees that use soil as a nesting material (e.g. mason bees), contact with residues of the neonicotinoid imidacloprid in soil may impair nesting activity and reproductive output [16,23,24], foraging behaviour [16], locomotion [25] and immature development [26].

Due to the mounting evidence of these negative effects on bees, the use of neonicotinoids has become more restricted [27] and new insecticides considered ‘safer for bees’ have entered the market. One of them, flupyradifurone (FPF), is a butenolide insecticide that works in a similar way to neonicotinoids (both are systemic, broad-spectrum and share the same mode of action) despite being from a different chemical class [28]. FPF is marketed as a ‘bee safe’ insecticide and has been assessed by the US Environmental Protection Agency (EPA) to be ‘practically non-toxic to adult honeybees' via contact exposure [29]. Consequently, unlike neonicotinoids, FPF can be used when a variety of crops are in bloom [29] and bees are most likely to visit the treated area.

Although oral toxicity for adult honeybees is acknowledged on the label of some FPF-based products [30], these labels often downplay the general toxicity to bees. Yet, despite claims of FPF being ‘bee-friendly’, field-realistic exposure to this novel insecticide can increase the risk of honeybee larval and worker mortality [31–34]. The few studies that have investigated the impact of FPF exposure on honeybees suggest sublethal effects comparable to those of neonicotinoids [28]. These include impairment of honeybee learning and cognition [32,35], motor activity [33,36], thermoregulation and flight success [37] and of foraging activity [38]. The alfalfa leafcutter bee (*Megachile rotundata*) appears to be 170-fold more sensitive to FPF than honeybees due to a lack of some insecticide-degrading P450 enzymes [39], but, to our knowledge, no study to date has tested the potential sublethal effects of FPF on any solitary bee species.

Bees in agricultural landscapes are often co-exposed to multiple pesticides, which may act synergistically to increase bee mortality [4,8]. Synergy manifests when combined pesticide effects surpass the predicted additive effect of individual compounds. Conversely, antagonistic effects occur when pesticides interfere, resulting in a lesser overall effect than the sum of individual responses. As a well-known example of synergism, sterol biosynthesis inhibitor (SBI) fungicides can inhibit cytochrome P450 detoxification in bees, increasing the toxicity of neonicotinoid and pyrethroid insecticides following co-exposure [2,4]. Recent evidence points to similar interactions between SBI fungicides (propiconazole and tebuconazole) and FPF [29,31], a combination that can also increase the frequency of abnormal behaviours (e.g. poor coordination, hyperactivity) in honeybees.

The solitary hoary squash bee (*Xenoglossa* (*Peponapis*) *pruinosa*) has recently been suggested as a model species to assess the effects of pesticides on ground-nesting bees [14,16]. In Ontario, Canada, adult squash bees emerge around July and remain active only for a few weeks, coinciding with bloom in *Cucurbita* crops [40]. Squash bees specialize on *Cucurbita* plants from which adult females collect pollen to provision brood cells in underground nests that they individually excavate. Each female produces a single generation of offspring per year that spend most of their life cycle developing underground until emergence during the following summer [41]. *Xenoglossa pruinosa* is associated with *Cucurbita* crops all across North America [14,42] and can nest directly in *Cucurbita* crop fields, making pesticide residues in soil a threat to their health and survival. Moreover, the absence of wild *Cucurbita* throughout most of *X. pruinosa*'s modern range [43] intensifies its reliance on agriculture. Neonicotinoid exposure has been found to decrease nest initiation, pollen harvesting and offspring production in squash bees [16], raising questions over whether FPF exposure has similar effects.

Here, we assessed the possible lethal and sublethal effects of realistic chronic exposure to two pesticides used on *Cucurbita* crops, the insecticide Sivanto Prime (FPF) and the fungicide Quadris Top (azoxystrobin and difenoconazole), either alone or combined, on the solitary ground-nesting hoary squash bee. Sivanto is used to protect cucurbit crops against aphids and whiteflies and can either be sprayed or applied directly to soil through irrigation water. FPF is persistent in soil [29], making its use particularly concerning for ground-nesting bees. Quadris Top protects cucurbit crops against common fungal diseases. The formulation combines the strobilurin fungicide azoxystrobin and the SBI fungicide difenoconazole, both of which are systemic and common contaminants of pollen, nectar and soil [2,19]. Although tank mixtures of Sivanto and Quadris Top are not permitted when applied to flowering crops according to label guidelines, both products can be used on the same crops during the same growing season, making co-exposure for bees highly probable. Indeed, Californian pesticide usage data (the only U.S. State for which such data are available) indicate simultaneous use on squash and other cucurbit crops [44].

Given the shared mode of action of neonicotinoids and FPF, and the potential for SBI fungicides to increase neonicotinoid toxicity, we predicted that (1) Sivanto will induce similar, though less pronounced, sublethal impacts on squash bees to those associated with neonicotinoid exposure (including reduced pollen harvesting and reproductive output [16] and heightened motor activity [36]), and (2) Quadris Top exposure will increase some of the Sivanto-induced effects on squash bees, indicating synergistic interactions between these two pesticide formulations.

## 2. Methods

### (a) Study design

We performed a semi-field experiment, using 10 hoop houses (W × L × H: 4.88 × 6.10 × 3.05 m) covered with bee-proof mesh and divided in the middle by a flexible wall made from transparent, colourless polyethylene plastic sheeting to obtain 20 experimental units (W × L × H: 4.88 × 3.05 × 3.05 m; electronic supplementary material, figures S1, S2*a,b*). Each plastic wall was buried at least 60 cm into the ground and acted as a barrier to prevent squash bees from crossing from one side of the hoop house to the other. The hoop houses were set up at a study site in Lakefield, Ontario, Canada in 2020 and were planted with untreated acorn squash seeds (variety Celebration in 2020 and Festival in 2021) in early June, at a density of twelve plants per enclosure (four rows of three plants).

Hoop houses were divided into three blocks to account for a soil texture gradient of increasing sand from North to South (electronic supplementary material, figure S1). Soil at the study site provided an excellent substrate for growing squash and for female squash bees to construct their nests [16]. No pesticide application occurred at the site during the year before the study (2019).

Treatments were assigned randomly following a split-plot design where the insecticide treatment (Sivanto or control) was applied to the full hoop house (i.e. main plot) and the fungicide treatment (Quadris Top or control) was applied to each half of the hoop house (i.e. subplot). There were five subplots (hereafter called ‘enclosures’) per treatment (*n* = 4: Sivanto, Quadris Top, Sivanto + Quadris Top, Control; electronic supplementary material, figure S1).

### (b) Pesticide applications

On 30 July 2020 (i.e. one week before squash bee introduction into enclosures), the insecticide Sivanto Prime was diluted in water (2 ml Sivanto l^−1^ water) and applied at the maximum label application rate (2000 ml ha^−1^) using plastic bottles with a hole in the lid that mimicked a drip irrigation system with a flow rate of 0.5 US gallons h^−1^ (or 1.89 l h^−1^; electronic supplementary material, figure S2*c*). The insecticide solution (or water alone for untreated plots) was applied at the base of each plant (125 ml plant^−1^) with additional water to ensure incorporation into the root zone.

To simulate agronomic use under a scenario of high disease pressure, three spray applications of Quadris Top (1.5 ml l^−1^ water) were made during the study, each at 7 day intervals. The first application was made in the early morning on 7 August 2020 (one week after the Sivanto application), 1 h before the introduction of squash bees into the hoop houses. The fungicide was sprayed at the maximum label rate (1000 ml ha^−1^), using an 8 l hand pump sprayer (SureSpray, Chapin), which was calibrated before application. Water alone was sprayed in the ‘untreated’ subplots, using a different sprayer of the same make and model. No pesticide was applied in 2021.

### (c) Squash bee introduction

Mated female squash bees were collected from a well-established nesting aggregation on a conventional pumpkin farm near Guelph, Ontario. Only females entering a nest with pollen on their scopa (pollen-carrying hairs on their hind legs) were collected to ensure these were mated [16]. Each bee was individually placed in a 2 ml centrifuge tube with an aeration hole and kept in a cooler with ice during transport. Upon arrival at the laboratory, each of the 130 bees collected was marked on their thorax using non-toxic waterproof Craft Smart paint pens of 10 different colours (electronic supplementary material, figure S2*d*) and transferred to a refrigerator where they were kept in the dark at 5°C overnight. The following morning, five marked female squash bees were released in each enclosure. Different colour markings were used for each side of the same hoop house. Of the 100 bees released, eight did not survive the stress of capture, transport and release into hoop houses (i.e. were dead after 24 h) and were replaced by new ones 2 days later. Data were collected for three weeks following squash bee introduction.

### (d) Survival

Survival was assessed through visual observations of individual marked bees. Every 1–3 days, observers spent a minimum of 10 min in each enclosure when bees were active (6.30–11.00) and recorded the presence of all bees inside flowers, in flight, or on the ground, leaves or other surfaces. The latest date on which each bee was seen was recorded as their presumed date of death.

### (e) Nesting activity

Each enclosure was surveyed for 40 min, three times a week, for active nests (i.e. when bees were observed entering the nest with pollen; electronic supplementary material, figure S3*a*). On each observation day, enclosures were randomly assigned between two observers who identified and marked each new active nest. The order in which enclosures were surveyed was also assigned randomly and all observations were made between 6.30 and 11.00. At the end of the season, we recorded the total number of marked nests per enclosure, as well as the exact location of each nest by measuring distances from the hoop house frames. Nest location was used to track offspring emergence during the following season (summer 2021).

### (f) Foraging activity

The number of pollen grains remaining on anthers of squash flowers after a single bee visit was used as a proxy for pollen collection. Studies show consistency in the number of pollen grains per anther between *Cucurbita* plants from the same varieties and across years [45,46].

Data on pollen collection by individual bees during single flower visits were collected a total of four times, on 13, 15, 20 and 26 August 2020. In each enclosure, two staminate (male) flowers were bagged with mesh netting 1 day prior to opening to prevent bee visitation. On the following morning, bags were removed and a single bee was allowed to visit each flower (electronic supplementary material, figure S3*b*). The duration of pollen collection (handling time) was recorded and, after the bee left the flower, the anther was removed and placed in a 2 ml microcentrifuge tube with 0.5 ml of 70% ethanol to quantify the number of unharvested pollen grains. If no bee entered the open flower after 10 min, the mesh bag was replaced, and a second attempt was made later that same morning. This was done a maximum of two times, after which the flower was discarded.

Back in the laboratory, each anther sample was centrifuged at 2500 r.p.m. for 3 min to dislodge pollen, after which the anther was removed. Each microcentrifuge tube was topped up to 1.5 ml by adding 1 ml of 50% glycerin solution and the sample was then thoroughly mixed with a mini-vortex mixer [16]. Four 5 µl aliquots were immediately taken out of the suspension and the numbers of pollen grains in each aliquot were counted on a grid under a microscope at 20× magnification. The mean number of pollen grains per aliquot was then related back to the full suspension volume.

### (g) Motor activity

We assessed the motor activity of squash bees two weeks after their introduction to the enclosures. To do this, we caught all bees that we could find in each enclosure on two consecutive mornings (22–23 August 2020) and placed them individually in transparent plastic tubes marked with five equally distanced parallel lines (electronic supplementary material, figure S3*c*). Bee activity was immediately video recorded for 2 min, after which the bees were released. Videos were later analysed to count the number of line crossings made in 2 min and the time bees spent engaging in grooming or escaping behaviours. A total of 56 videos of individual bees were recorded (34 on day 1, 22 on day 2).

### (h) Crop yield and flower counts

For 5 days, between 15 August and 20 August, we marked all pistillate (female) flowers within each enclosure with flagging tape under the undeveloped fruits (ovary; electronic supplementary material, figure S3*d*). At the end of the season (19 September 2020), each ovary was characterized as either aborted or having set fruit, and all fruits were harvested and weighed. We then calculated the percentages of fruit set and marketable fruits (≥ 500 g) for each enclosure [16]. To account for the effect of food availability on the different response variables assessed, we counted the total number of pistillate and staminate flowers (electronic supplementary material, figure S2*e*) in each enclosure on 13 different days over the three weeks of the experiment and calculated a mean number of flowers per day.

### (i) Offspring production

In 2021, total offspring production was assessed by collecting all bees that emerged in each enclosure daily, except during weekends. Upon emergence, unmated female and male hoary squash bees typically rest in wilted *Cucurbita* flowers during the afternoon and night [41]. Knowing this, every afternoon from mid-July to early September we checked all wilted flowers for the presence of squash bees. The first offspring emerged on 30 July and, as such, the number of days after 29 July was used as the response variable to assess the effect of treatments on the date of offspring emergence. Newly emerged squash bees were sexed and immediately frozen. We then measured the intertegular (IT) span, or distance between wing bases, of each bee as an indicator of body size, using digital calipers under a dissecting microscope.

Emergence tents (BugDorm BT2006; electronic supplementary material, figure S3*e*) were also used to assess offspring production per individual nest. The tents (two per enclosure, except for enclosures 1CB and 1AA where only one tent could be installed) were installed directly on top of marked active nests from the previous season. We only selected nests that were isolated enough from each other to avoid overlap. As with total offspring production, we checked each tent daily for the presence of newly emerged bees. We added ethanol (70%) to the collecting jar only during weekends, when daily observations were not possible. For offspring trapped during the weekend (Saturday to Monday), the following Monday was considered as the date of emergence. All bees caught in emergence tents were included in the total number of offspring that emerged per enclosure and their IT spans were measured as described above.

### (j) Pesticide residues

To assess pesticide residue concentrations in crop soil, nectar and pollen, we collected samples of each matrix multiple times during the experiment. Soil samples (0–15 cm depth) were collected from each enclosure at the beginning (6 August 2020) and end of the experiment in 2020 (27 August 2020), and in August of the following season (2 August 2021). Seventeen soil cores were collected throughout the planted area (electronic supplementary material, figure S4) and thoroughly mixed to obtain one 50 g subsample per enclosure.

In 2020, squash pollen and nectar were collected directly after the first and second fungicide applications (7 and 14 August), as well as 2 and 5 days after the first fungicide application (9 and 12 August) and on the last day of the experiment (28 August). Pollen and nectar were not collected in 2021. One staminate flower per enclosure was bagged with mesh netting on the day prior to petal opening to prevent pollen and nectar collection by bees. Open flowers were then picked and nectar was collected using a 20 µl microcapillary pipette. Pollen was removed from the stamen using a plastic knife. Pollen and nectar of flowers sampled from enclosures of the same treatment group were pooled in 2 ml centrifuge tubes. Nectar was also collected from pistillate flowers on the last day of the experiment to assess potential differences in pesticide residues between staminate and pistillate flowers.

All soil, nectar and pollen samples were kept at −20°C until analysis. Frozen samples were extracted using the EN 15662 QuEChERS procedure and screened for pesticide residues by liquid chromatography–mass spectrometry (LC–MS/MS) at the Chemical Ecology Core Facility at Cornell University (Ithaca, NY, USA) (electronic supplementary material, appendix S1).

### (k) Data analysis

Of the 100 bees released into hoop houses, three were found to have crossed from one side of a hoop house to the other and were excluded from the analysis for survival, motor activity and foraging activity. Because this resulted in an unequal number of bees in the enclosures involved (1AA, 1AB, 3CA, 3CB), these were excluded from the analysis for number of nests, crop yield and total number of offspring. As a result, the final number of enclosures per treatment for these analyses were as follows: control (*n* = 5), Sivanto (*n* = 3), Quadris (*n* = 5), Sivanto + Quadris (*n* = 3). Alternative analyses that include all enclosures while correcting for the unequal number of bees per experimental unit are presented in electronic supplementary material, appendix S2. In addition, it should be noted that we observed more female squash bees than released (i.e. six instead of five) in two additional enclosures (2BB, 2CA). These unmarked females were observed for the first time during the last week of the experiment and most likely emerged from underground nests from the previous season. Considering that these additional female squash bees were likely unmated (there were no males to mate with in the enclosures) and emerged late in the experiment, enclosures 2BB and 2CA were retained in all analyses.

All statistical analyses were performed in R v. 4.3.1 [47]. Bee survival among treatment groups was assessed using a logrank Kaplan–Meier survival analysis (survival package in R). Bees that survived until the end of the experiment (21 days) were right-censored. All other data were analysed using either linear mixed models (LMMs) or generalized linear mixed models (GLMMs), depending on the probability distribution of each independent variable. Models were fitted using the lme4 package, except those requiring negative binomial or beta distributions, which were fitted using the glmmTBM package [48] (see electronic supplementary material, table S2 for details and references). Model error distributions were determined based on the nature of each response variable and compliance with model assumptions (electronic supplementary material, table S2). The following fixed effects were included in models: insecticide treatment (all models), fungicide treatment (all models), date of data collection (for motor activity and pollen collection), sex of newly emerged bees (for offspring IT span and offspring emergence date), as well as all two- and three-way interactions between these variables. To take into account the potential effect of floral resource availability on nesting activity, foraging activity, motor activity and offspring production, we included the average number of pistillate and total flowers as potential covariates. For models assessing the potential effects of pesticide treatments on foraging and motor activity, we also included categorical covariates of the date of squash bee introduction and the time of data collection (early, mid or late morning). Finally, the total duration of time that bees spent in grooming or escaping behaviour was also included as a covariate in the model for motor activity. Considering the relatively small sample size per group resulting from dividing the dataset for motor activity by sampling date, we also fitted an alternative model that excluded the interaction between date of sampling and the pesticide treatments (see electronic supplementary material, Appendix 2). In all models, the blocking effect of soil texture was included as a random variable, as well as the main plot factor (insecticide treatment), nested within blocks, to account for the structure of the experimental design (split-plot). The dates of data collection and all covariates were considered only if inclusion improved the model fit (based on lowest AICc values), while the random variables were retained in all models (electronic supplementary material, table S2). Because paint markings started to fade with time, it was not always possible to determine the identity of individual bees used in trials on pollen collection and motor activity. As such, individual bee ID was not included as a random effect in models (i.e. data were not treated as repeated measures).

Adherence to the appropriate assumptions was tested with diagnostic plots and tests. Significance of the modelled terms was assessed using type III analysis of variance (ANOVA) with Satterthwaite approximation for degrees of freedom, and significant interaction effects were decomposed by fitting simple two-way interactions and simple main effects. In cases of a significant interaction between pesticides, a Tukey–Kramer *post hoc* test was performed to assess differences in responses between the co-exposed group and the control. Significance was defined as *p* ≤ 0.05 for all analyses.

To determine whether interactions between pesticides had an overall synergistic, antagonistic, or additive effect, we calculated interaction effect sizes for each response variable, using Hedges' *d* values following [49] (see electronic supplementary material, appendix S3 for details). Interactions denoted as possible synergism when the effect size (Hedges’ *d* value) was positive and the 95% confidence interval (CI) did not include zero, antagonism when the effect size was negative and the 95% CI did not include zero, and additivity when the 95% CI included zero. Note that, in the context of our study, these results suggest potential non-additive interactions rather than definite synergism or antagonism. Indeed, there is currently no general agreement on the statistical determination of synergy for treatment interaction at specific doses (i.e. without a dose–response relationship) for the range of sublethal endpoints assessed in our study [50,51]; and while Hedges' *d* values are a useful tool for evaluating deviation from additivity, the method, as many others, lacks statistical testing to account for variation of response to treatment [50].

## 3. Results

### (a) Effects of pesticide exposure on bee survival and nesting activity

Squash bees were observed digging nests as early as one day after being released. A total of 80 active nests (mean ± s.d.: 4.0 ± 1.9 per enclosure) were marked across the 20 enclosures during the three weeks of observations. Sixty-two female squash bees (62%) were still alive two weeks after introduction in enclosures and 24 bees (24%) survived until the end of the experiment. We found no effect of pesticide treatments on the survival of female squash bees (logrank, *p* = 0.400; electronic supplementary material, figure S5) or the number of nests they established (electronic supplementary material, tables S2–S3).

### (b) Effects of pesticide exposure on bee foraging and motor activity

**Figure 1.**
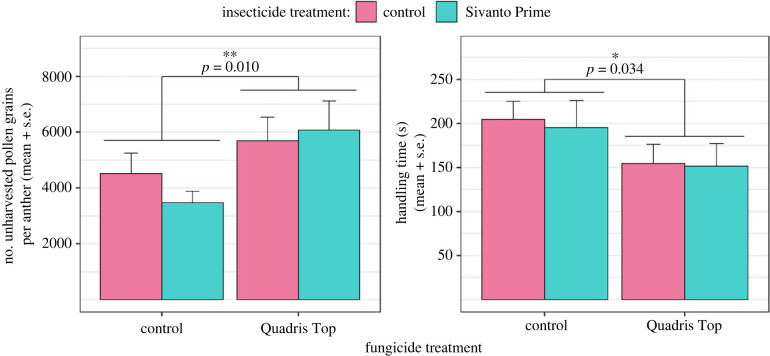
Effect of pesticide treatments on pollen collection (number of pollen grains left on anthers) and flower handling time by individual female squash bees (*n* = 47; 10–13 per treatment group) during single flower visits.

On average, female squash bees collected 32% less pollen per single flower visit (*F*_1,40_ = 7.394, *p* = 0.010) and spent 24% less time handling squash flowers (*F*_1,40_ = 4.848, *p* = 0.034) in enclosures treated with Quadris Top (figure 1). We found no effect of Sivanto or the interaction between Sivanto and Quadris Top on pollen collection (number of pollen grains left on anthers and handling time of flowers) by squash bees.

**Figure 2.**
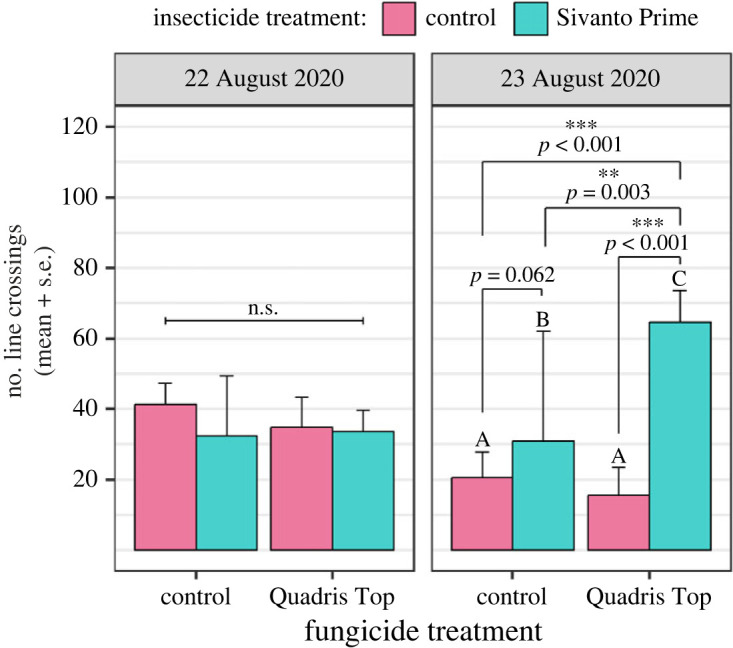
Effect of pesticide treatments on the motor activity (number of line crossings in 2 min) of female squash bees. Data were collected during two consecutive days, on 22–23 August 2020. Different letters represent significantly different responses (Tukey–Kramer).

**Figure 3.**
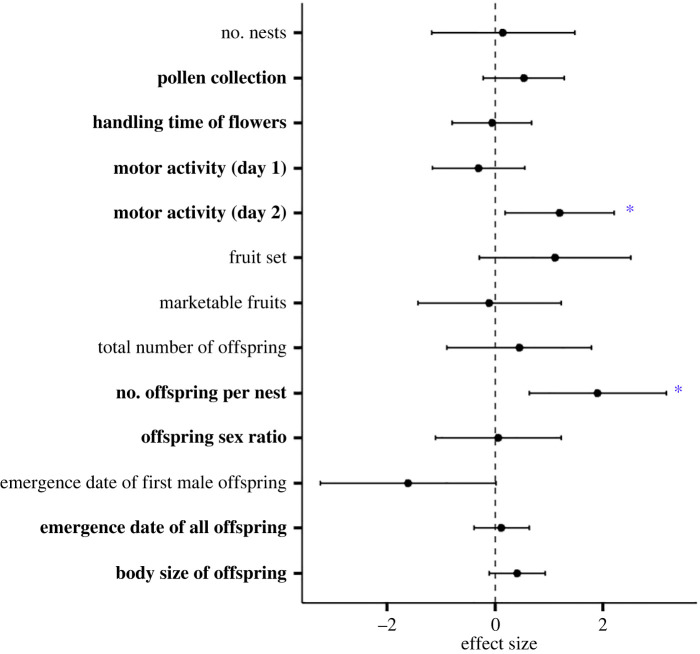
Interaction effects (Hedges' *d* value ± 95% CI) of pesticide treatments on all response variables measured. Interactions are synergistic (*) when the effect size is positive and the 95% CI does not include zero, antagonistic when the effect size is negative and the 95% CI does not include zero, and additive when the 95% CI includes zero. The number of enclosures included in analyses varies by endpoints: bold text = full factorial design, plain text = some enclosures were excluded (see electronic supplementary material, table S1 for details).

We found a significant triple interaction between the two pesticide treatments and the day of data collection on squash bees' motor activity (*F*_1,43_ = 6.597, *p* = 0.014; figure 2). Specifically, there was a statistically significant two-way interaction between pesticide treatments (*F*_1,15_ = 41.732, *p* < 0.001), but only on the second day of data collection (23 August). Computing simple main effects for each pesticide treatment revealed that, on 23 August, the combined exposure to both pesticides induced hyperactivity in squash bees (figure 3). Indeed, the number of line crossings recorded was significantly higher for bees that were exposed to both pesticides compared to those that were only exposed to either Sivanto (*F*_1,9_ = 55.412, *p* < 0.001) or Quadris Top (*F*_1,11_ = 167.295, *p* < 0.001) or that were unexposed (Tukey–Kramer: *p* < 0.001; electronic supplementary material, table S7). Similarly, excluding interactions between sampling date and pesticide treatments from the model to increase sample size (electronic supplementary material, appendix S2) resulted in a significant interaction between pesticide treatments on bee motor activity (*F*_1,46_ = 4.630, *p* = 0.037; electronic supplementary material, table S2), such that the bees were more active following a combined exposure to both pesticides. The Hedges' *d* values calculated suggest that the combined effect of pesticides on bee motor activity could be synergistic (electronic supplementary material, table S8).

### (c) Effects of pesticides on crop yield

A total of 187 pistillate flowers were marked across the 20 enclosures, of which 68 (36.4%) set fruits (7–100% per enclosure) and 40 (21.4%) produced fruits of marketable weight (greater than 500 g). The random effect of block significantly impacted the percentage of marketable fruits, which increased with decreasing percentages of sand in soil (mean percentage of marketable fruits ± s.d.: 15.0 ± 12.2% in block A, 23.8 ± 20.6% in block B, 50.2 ± 32.5% in block C). We found no effect of pesticide treatments on either fruit set or marketable squash yield (figure 3; electronic supplementary material, tables S2–S3).

### (d) Effects of pesticide exposure on bee offspring production

**Figure 4.**
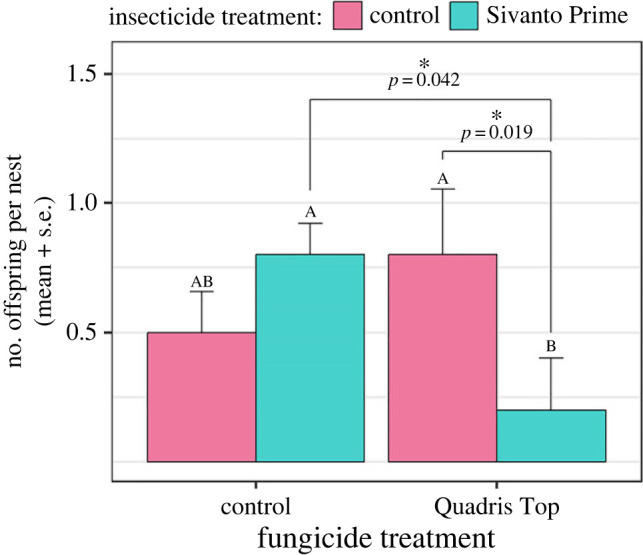
Effect of pesticide treatments on the mean number of offspring that emerged in 2021 from active nests established by female squash bees in the previous season. Emergence tents (*n* = 78) were installed above individual marked nests and offspring emergence was assessed daily from mid-July to early September. Different letters represent significantly different responses (Tukey–Kramer).

A total of 99 offspring squash bees emerged across the 20 enclosures in 2021, ranging from 0 to 15 per enclosure (mean ± s.d.: 5.0 ± 3.7 offspring). The first offspring emerged on 30 July and the last on 2 September. Although the total number of emerged offspring per enclosure did not significantly differ among treatments (electronic supplementary material, tables S2–S3), we found a significant interaction between Sivanto and Quadris Top on the mean number of offspring per nest (*F*_1,14_ = 5.823, *p* = 0.029; electronic supplementary material, table S2). Computing simple main effects of pesticide treatments revealed that the mean number of offspring that emerged per nest was significantly lower for bees that were exposed to both pesticides compared to those that were only exposed to either Sivanto (*F*_1,6_ = 8.208, *p* = 0.028) or Quadris Top (*F*_1,6_ = 4.966, *p* = 0.064; marginal significance; electronic supplementary material, table S6). Although the Hedges' *d* value suggests that this interaction could be synergistic (figure 3), the difference observed among treatments was modest (figure 4) and results from the Tukey–Kramer test revealed no significant difference in response between the mixture treatment and the control (*p* = 0.292; electronic supplementary material, table S7). We also found a significant interaction between both pesticides (and a trend for an antagonistic response of combined pesticide exposure, based on the Hedges' *d* value; figure 3; electronic supplementary material, table S8) on the date of first male offspring emergence (*F*_1,5_ = 12.343, *p* = 0.017). Because male squash bees emerge earlier than females [41], only the enclosures (*n* = 12) in which the first emerged offspring was male were used to assess the effect of treatments on the date of first offspring emergence. The first male offspring emerged significantly earlier in enclosures treated with Sivanto (*F*_1,2_ = 86.712, *p* = 0.011), but only when Quadris Top was not applied (electronic supplementary material, figure S6), such that Quadris Top overshadowed the effect of Sivanto. However, no treatment effect was found when the emergence dates of all 99 offspring were considered (electronic supplementary material, table S2). Exposure to Sivanto resulted in slightly larger offspring (electronic supplementary material, table S8), independently of the fungicide treatment (*F*_1,89_ = 5.974, *p* = 0.016; electronic supplementary material, figure S7). No effect of treatment was found on the sex ratio of emerged offspring (number of males ÷ total number of offspring), which averaged 0.35 ± 0.24 (mean ± s.d.) among all enclosures. No male emergence (only female) was recorded in four of the 19 enclosures in which offspring emerged.

### (e) Pesticide residues in soil, pollen and nectar

In enclosures treated with Sivanto, residue concentrations of FPF in soil ranged from 168 to more than 1000 ng g^−1^ at the beginning of the experiment, 307 to more than 1000 ng g^−1^ at the end of the experiment and 7–476 ng g^−1^ in the following summer (August 2021; electronic supplementary material, table S9). No FPF residues were found in soil from untreated enclosures at any sampling time. Residues of azoxystrobin (101–392 ng g^−1^) and difenoconazole (134–473 ng g^−1^) were also present in soil within fungicide-treated enclosures and rarely moved to adjacent subplots. Trace residues of other agrochemicals (e.g. atrazine, clothianidin) were also detected in soil in most hoop houses, likely resulting from previous use of treated seeds at the research site or contamination from nearby agricultural fields (full list of active ingredients available at [52]).

FPF translocated from soil to squash nectar and pollen, with residue concentrations averaging 42.52 ± 25.55 ng g^−1^ (mean ± s.d.) in nectar and 32.32 ± 25.37 ng g^−1^ in pollen throughout the experiment (electronic supplementary material, table S10). Both azoxystrobin and difenoconazole were detected in nectar (the highest concentration measured was higher than the quantification limit of our analytical methods, which was 3333 ng g^−1^) and pollen (up to greater than 7000 ng g^−1^) from treated squash plants, with concentrations gradually decreasing from the time of application to up to 5 days post-application (electronic supplementary material, table S10). Pooled samples of nectar and pollen collected from squash flowers in unsprayed enclosures sometimes contained very low concentrations of both fungicides (electronic supplementary material, table S10). No residues of pesticides other than those applied in our study were retrieved in squash nectar and pollen.

## 4. Discussion

Chronic single and combined exposure of ground-nesting squash bees to pesticides used on squash crops resulted in effects on pollen collection, motor activity, reproductive output, timing of offspring emergence and offspring body size that could affect squash bee populations in multiple ways. Some of these effects were either induced by the fungicide Quadris Top (reduced pollen collection) or the insecticide Sivanto Prime (increased offspring body size), while others were revealed as interactions between the two pesticides, pointing to mechanisms that were possibly synergistic (hyperactivity) or antagonistic (early emergence of male offspring following exposure to Sivanto, which was suppressed by co-exposure to Quadris Top). Compared to single pesticide exposure, co-exposure to both pesticides also resulted in a lower number of emerged offspring per nest, although this response was not significantly different from the control group. This study demonstrates the potential of pesticides with low acute toxicity, such as fungicides and novel ‘bee-safe’ insecticides, to adversely affect squash bees when used at field-realistic application rates, whether applied alone or in combination.

### (a) Behavioural effects of pesticide exposure on squash bees

Spraying fungicides on squash plants decreased per-flower pollen collection by female squash bees. The effect was not influenced by the day of data collection, suggesting that systemic residues translocated into pollen throughout the experiment. This was confirmed by the detection of residues (more than 300 ng g^−1^) in squash pollen collected up to 5 days after fungicide application. A similar decrease in cranberry pollen collection by honeybees occurred following application of the SBI fungicide prothioconazole to cranberry bogs, but not azoxystrobin [53], suggesting that difenoconazole (also an SBI fungicide) may be responsible for the negative behavioural response observed here.

Reduced pollen collection could lead to slower gathering of food provisions and nest cell production rate by the mother squash bees or the allocation of fewer pollen resources to each offspring, which could further influence the immature development, adult body size and overall fitness of the offspring [23]. Impaired foraging efficiency may also result in a shift towards production of male offspring, which require smaller pollen provisions in order to develop [23]. However, nest establishment and offspring production (including offspring body size and sex ratio) appeared unaffected by the main effect of fungicide exposure, suggesting that provision size was not affected. To compensate for shorter individual flower visits, female squash bees may have visited more flowers overall. Where floral contamination is heterogeneous, this strategy may represent an adaptive behaviour that increases the likelihood of finding uncontaminated pollen and may bring benefits to both the mother bee and offspring by decreasing fungicide exposure. It is possible that smaller pollen provisions interacted with contact exposure to FPF residues in soil during immature development, resulting in lower numbers of offspring per nest reaching adulthood in enclosures treated with both pesticides. However, more research is needed to determine whether this interactive effect of pesticide treatments on offspring emergence is related to food deprivation, impaired detoxification mechanisms leading to increased toxicity and potential higher mortality rate of immature bees, or a combination of these factors.

Combined pesticide exposure induced hyperactivity in squash bees, a behavioural response that was also influenced by time (day of data collection). Like neonicotinoids, FPF stimulates acetylcholinergic neurons and can lead to increased activity in the central nervous system and motor neurons [31,37]. This hyperactivity could impair flight performance, foraging and nest-building abilities [25,31,37], leading to a decrease in nest provisioning efficiency. We indeed found a reduced number of offspring per nest in enclosures treated with both pesticides. Here, the effect of FPF was amplified by co-exposure with Quadris Top, suggesting possible inhibition of insecticide detoxification by one (or both) of the fungicide active ingredients. Considering the known potential of SBI fungicides to increase negative effects of neonicotinoids and FPF in bees, difenoconazole is likely driving this synergistic response. Further, the time component of the interaction (i.e. synergism observed only on day 2) can be explained by the timing of fungicide application. Videos of motor activity were recorded 24 h and 48 h after the last fungicide application. Delayed and time-cumulative toxic effects of pesticide exposure, including FPF [54], neonicotinoids [55] and fungicides [56], are common for honeybees and may reflect an accumulation of residues in the bee body and/or a delayed impairment of detoxification mechanisms.

### (b) Effect of pesticide exposure on squash bee offspring production

The observation that the total number of offspring per enclosure was not affected by pesticide exposure while the mean number of offspring per nest was lower for co-exposed bees compared to those exposed to single pesticides suggests that the negative impacts might have been concentrated within the nest provisioning and brood care behaviours of individual bees. This could be attributed to synergistically induced hyperactivity. These changes may lead to suboptimal larval development and reduced nest-level reproductive success, despite the population maintaining its overall reproductive output. The absence of a population-level effect hints at complex and adaptive population responses and potential pesticide sensitivity variation among bees. It is worth noting that while no statistically significant effect of pesticide treatments was found on the total number of offspring per enclosure, a trend towards reduced total offspring production was observed when both pesticides were applied (electronic supplementary material, table S8). Differences between nest-level and population-level outcomes could be influenced by reduced statistical power in the latter analysis due to smaller sample size. Further research is needed to confirm these explanations and explore underlying mechanisms.

Our results suggest that low (field-realistic) levels of FPF may lead to favourable biological responses for ground-nesting bees. Indeed, the early emergence and increased body size of squash bee offspring in enclosures treated with Sivanto may reflect possible hormetic effects, whereby exposure to low (sublethal) doses of a substance stimulates biological processes, leading to fitness benefits, while exposure to high doses decreases fitness [57]. However, having used only a single application rate per pesticide, our experimental design did not allow us to test for such effects. The impact of FPF on offspring body size could also be explained by a potential survival bias favouring larger individuals, although it is important to note that offspring count was not affected by the direct effect of FPF exposure. In solitary bees, intra-specific variation in bee size is related to movements [25] and may influence foraging, floral handling and pollination services at ecosystem scales [58]. Moreover, timing of emergence has the potential to alter population dynamics as male squash bees that emerge earlier may be more likely to mate with more receptive females in their lifetime [40].

Interestingly, our results suggest a possible antagonistic interaction between Sivanto and Quadris Top on the emergence date of squash bee offspring, where fungicide exposure suppresses early emergence of male offspring. It is worth noting that the statistical significance of this effect is based on a sample size of two enclosures for the Sivanto treatment (electronic supplementary material, figure S6), and care should be taken when interpreting this result. Although not as common as synergism, there are many examples of antagonistic responses of bees to combined exposure to agrochemicals [4,59]. In addition to inhibiting P450 detoxification in bees, SBI fungicides may also induce P450 enzyme activity at very low levels of exposure, thus improving detoxification [59]. This highlights, once again, the complexity of potential interactions between agrochemicals.

### (c) Pesticide exposure and environmental considerations

As FPF was detected in squash pollen and nectar as well as in soil, the effects of Sivanto on squash bee offspring (i.e. earlier emergence and larger size) could have resulted from an exposure of immature bees through any of these matrices. Unfortunately, our results do not allow us to determine whether exposure of immature bees occurred mostly via contact with residues in soil or via the consumption of (or contact with) contaminated pollen. Compartmentalizing the different routes of exposure by providing bees with either contaminated soil or contaminated food sources, but not both, would be a way to address this question in future studies.

The 2020 growing season was hot and dry in Ontario, which might have affected our results in various ways. For instance, moist soil seems to stimulate digging and nesting activity by ground-nesting bees [60], possibly by making soil easier to excavate. The toxicity of pesticide residues to soil arthropods may also be influenced by the degree of soil moisture, as reported for *Osmia lignaria* [61]. Indeed, soil moisture likely increases pesticide bioavailability to insects, potentially making residues in wet soil more hazardous to ground-nesting bees. Whether effects of Sivanto exposure may be more pronounced than reported in this study during wet years as well as for ground-nesting bees nesting in irrigated fields remains to be investigated.

While exposure to all three active ingredients occurred via both soil and food resources, concentrations of FPF were the highest in soil and concentrations of azoxystrobin and difenoconazole were the highest in pollen and nectar. Residue concentrations of FPF in our study were lower than those detected in the pollen and nectar collected by honeybees foraging on melon fields [29], suggesting that squash bees may routinely be exposed to higher concentrations than we used. Azoxystrobin and difenoconazole residue levels in our study fall within the range of concentrations previously reported for honeybee-collected pollen and honey [2], except on application days when concentrations were typically higher. The fact that low fungicide residue concentrations were detected in the nectar and pollen of flowers collected from unsprayed enclosures suggests that cross contamination between treated and untreated subplots may have occurred. However, contamination of untreated flowers with fungicides appeared to only be sporadic and residues were extremely low compared to those from treated flowers. As such, we believe this artefact to have only a minor influence on our results and their interpretation.

## 5. Concluding remarks

Reductions of over 85% in nest establishment and offspring production have been reported for female squash bees exposed to a squash crop treated at planting with the highly toxic neonicotinoid insecticide, imidacloprid [16]. Although not entirely comparable, our results suggest that exposure to FPF may have less dramatic effects than imidacloprid for female squash bees when applied to *Cucurbita* crops. Instead, the effects found in this study were more subtle, with complex interactions in play. Current pollinator risk assessments by the Environmental Protection Agency (US EPA) and the European Food Safety Authority (EFSA) do not consider contact exposure with soil residues, as honeybees do not generally have direct contact with soil, nor do they consider exposure to pesticide mixtures [9]. As such, many of the effects reported in this study, which may have important consequences for population dynamics of solitary ground-nesting bees, would be completely overlooked by these risk assessments. Our study raises concerns about the ecotoxicological profile of pesticides with low acute toxicity and highlights the need for more thorough pesticide risk assessments that also consider potential sublethal and interactive effects for ground-nesting bees and other wild bees, especially for novel compounds that are likely candidates to replace neonicotinoids. These concerns also extend to the diverse array of animals and plants for which a multitude of adverse effects stemming from neonicotinoid exposure have been documented.

In the face of regional and global species declines, it is imperative to evaluate the impacts of individual and combined field-realistic pesticide exposures on ecologically relevant endpoints. The development of field and semi-field ecotoxicological studies should be accompanied by the concurrent refinement of statistical methods for determining synergy between pesticides in real-world settings, notably on sublethal endpoints. This holistic approach is crucial for obtaining optimal results and advancing our understanding of the ecological implications of pesticide exposure.

## Data Availability

The data and R code that support the findings of this study are openly available in Dryad at: http://doi.org/10.5061/dryad.b2rbnzsp6 [52]. Supplementary material is available online [62].
